# Compressed Deep Learning to Classify Arrhythmia in an Embedded Wearable Device

**DOI:** 10.3390/s22051776

**Published:** 2022-02-24

**Authors:** Kwang-Sig Lee, Hyun-Joon Park, Ji Eon Kim, Hee Jung Kim, Sangil Chon, Sangkyu Kim, Jaesung Jang, Jin-Kook Kim, Seongbin Jang, Yeongjoon Gil, Ho Sung Son

**Affiliations:** 1 AI Center, Korea University Anam Hospital, Seoul 02841, Korea; ecophy@korea.ac.kr; 2 Institute for Health Service Innovation, Korea University College of Medicine, Seoul 02841, Korea; hyunjun21@korea.ac.kr; 3 Department of Thoracic and Cardiovascular Surgery, Korea University College of Medicine, Korea University Anam Hospital, Seoul 02841, Korea; jieonkim82@gmail.com (J.E.K.); heejung440@daum.net (H.J.K.); 4 HUINNO Co., Ltd., Seoul 06011, Korea; chons@huinno.com (S.C.); kimsk6015@huinno.com (S.K.); jaeseongjang@huinno.com (J.J.); jinkook@huinno.com (J.-K.K.); sbjang@huinno.com (S.J.); kyzoon@huinno.com (Y.G.)

**Keywords:** arrhythmia, compressed deep learning, embedded wearable device, Resnet, Mobilenet

## Abstract

The importance of an embedded wearable device with automatic detection and alarming cannot be overstated, given that 15–30% of patients with atrial fibrillation are reported to be asymptomatic. These asymptomatic patients do not seek medical care, hence traditional diagnostic tools including Holter are not effective for the further prevention of associated stroke or heart failure. This is likely to be more so in the era of COVID-19, in which patients become more reluctant on hospitalization and checkups. However, little literature is available on this important topic. For this reason, this study developed efficient deep learning with model compression, which is designed to use ECG data and classify arrhythmia in an embedded wearable device. ECG-signal data came from Korea University Anam Hospital in Seoul, Korea, with 28,308 unique patients (15,412 normal and 12,896 arrhythmia). Resnets and Mobilenets with model compression (TensorFlow Lite) were applied and compared for the diagnosis of arrhythmia in an embedded wearable device. The weight size of the compressed model registered a remarkable decrease from 743 MB to 76 KB (1/10000), whereas its performance was almost the same as its original counterpart. Resnet and Mobilenet were similar in terms of accuracy, i.e., Resnet-50 Hz (97.3) vs. Mo-bilenet-50 Hz (97.2), Resnet-100 Hz (98.2) vs. Mobilenet-100 Hz (97.9). Here, 50 Hz/100 Hz denotes the down-sampling rate. However, Resnets took more flash memory and longer inference time than did Mobilenets. In conclusion, Mobilenet would be a more efficient model than Resnet to classify arrhythmia in an embedded wearable device.

## 1. Introduction

Heart disease is a major contributor for disease burden on the globe [1,2,3,4,5,6]. The estimated number of deaths from cardiovascular disease was 17.9 million in the world for Y2019 (Y2019 hereafter), which was 32% of global deaths [1]. The age-standardized death rate from atrial fibrillation, the most common arrhythmia, showed a great increase from 0.8 to 1.6 per 100,000 for men (or 0.9 to 1.7 per 100,000 for women) in the world during 1990–2010 [2]. This worldwide trend agrees with its Korean counterpart. Heart disease ranked second in Korea as the cause of death for Y2020 (63.0 per 100,000) [3] and as the source of disease burden for Y2015 (3475 disease-adjusted life years per 100,000) [4]. In addition, the number of hospitalizations for atrial fibrillation registered a rapid growth of 420% from 767 to 3986 per 1 million during 2006–2015 [5].

For this reason, emerging literature has focused on the early diagnosis of arrhythmia, using deep neural networks for their better performance measures than those of other approaches [6,7,8,9,10,11,12,13,14]. These studies utilized electrocardiogram (ECG) data, applying convolutional neural networks (Alexnet, Resnet) [6,7,8,9,10,11,12], recurrent neural networks (long short-term memory) [13] or both [14] with various class categories and accuracy results (80–99%). For instance, a recent study [11] employed ECG data in a general hospital, comparing 30 convolutional neural networks for the classification of the normal sinus rhythm vs. atrial fibrillation status: six Alexnets with five convolutional layers, three fully connected layers and 3 to 256 kernels; and 24 Resnets with 2 to 8 residual blocks and 2 to 64 kernels. The accuracy of the best Alexnet was 0.997 with 24 kernels in the first layer, 5,268,818 parameters and the training time of 89 s, while the best Resnet showed the accuracy of 0.999 with six residual blocks, 32 initial kernels in the first layer, 248,418 parameters and the training time of 253 s. In general, the performance of Resnet improved as the number of its residual blocks (its depth) increased. Based on the results of this study, for atrial fibrillation diagnosis, Resnet might be a good model with higher accuracy and fewer parameters than its Alexnet counterparts.

A recent follow-up [12] made two extensions to the study above. In this follow-up, six types of arrhythmia were considered, i.e., atrial fibrillation, atrial flutter, sinus bradycardia, sinus tachycardia, premature ventricular contraction and first-degree atrioventricular block. This study also introduced Resnet with a squeeze-and-excitation block (SE-Resnet) and compared SE-Resnet to its baseline counterpart for varying layer depth (18, 34, 50, 101, 152). Based on the findings of this study, SE-Resnet outperformed its baseline counterpart across the board. Specifically, SE-Resnet with 152 layers showed the highest F1 score of 97.05% with a margin of 1.40% compared to its baseline counterpart. However, these models are reported to take too much memory for an embedded wearable device. The importance of an embedded wearable device with automatic detection and alarming cannot be overstated, given that 15–30% of patients with atrial fibrillation are reported to be asymptomatic [15,16,17]. These asymptomatic patients do not seek medical care hence traditional diagnostic tools including Holter are not effective for the further prevention of associated stroke or heart failure [18]. This is likely to be more so in the era of COVID-19, in which patients become more reluctant on hospitalization and checkup [19,20].

Resnet [21], Mobilenet [22] and Litenet [23] are deep learning candidates for embedded vision applications. Resnet is based on residual learning (to be explained in the next section). Residual learning brought it to the first place in 2015 ImageNet Large Scale Visual Recognition Challenge with 152 layers and top-5 error rate of 3.6%. Residual learning brought it to much greater depth and accuracy compared to Virtual Geometry Group (the second winner in 2014 with 24 layers and a top-5 error rate of 6.8%) [21]. Mobilenet [22] and Litenet [23] center on depth-wise and point-wise convolutions, which reduce the size of input image and the number of its channels, respectively. A recent study used Litenet to classify arrhythmia and achieved the accuracy of 97.78% in the inference time of 25 microseconds [23]. These deep learning models depend on the strengths of convolutional layers, which focus on global information. On the other hand, another group of models rely on the distinctive characteristics of recurrent layers, which focus on sequential information [24,25]. One recent study used a linear combination of simple recurrent neural networks for the diagnosis of arrhythmia, recording the accuracy of 99.60% in the inference time of 31.2 ms [24]. Likewise, another recent study requested due attention to the advantage of combining convolutional layers and simplest (Vanilla) recurrent layers for the diagnosis of arrhythmia, recording the accuracy of 99.80% in the inference time of 3 min [25]. However, the existing literature employed a public dataset (MIT-BIH Arrhythmia Database) and its inference was performed on personal computers, not in an embedded wearable device. In this context, this study introduced efficient deep learning with model compression, which is tailored for ECG data and arrhythmia classification in an embedded wearable device. To the best of our knowledge, this is the first study in this direction.

This article is organized in the following manner. Participants, deep learning models and their compression methods are described in the next section. This is followed by the presentation of their results in terms of performance, model size, inference time and current consumption. Finally, the contributions, limitations and conclusions of this study are discussed in the last section.

## 2. Materials and Methods

### 2.1. Participants and Categories

**Figure 1:**
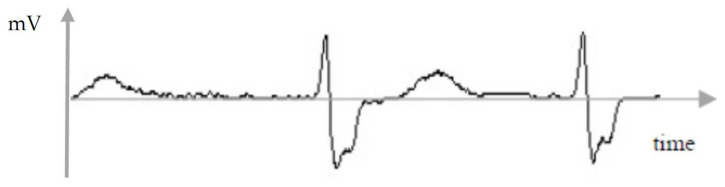
Electrocardiogram Signal.

ECG-signal data came from Korea University Anam Hospital in Seoul, Korea, with 28,308 unique patients. Other information including age, gender and medical history was excluded from this dataset because of hospital rules and regulations. This retrospective study was approved by the Institutional Review Board of Korea University Anam Hospital on 12 February 2018 (2018AN0037). Informed consent was waived by the IRB given that data were de-identified. Lead-II ECG-signal data (taken from 12-lead ECG image traces) were measured for 10 s at the frequency of 200 Hz. Among the 28,308 patients, 80%, 10% and 10% were used as training, validation and test sets, respectively. Training/validation was performed in a personal computer whereas testing was completed on an embedded wearable device. Among the 28,308 patients, 15,412 were diagnosed as normal (Categories 1–4 in Table 1) and 12,896 as arrhythmia (Categories 5–7 in the table). A normal ECG wave has five elements: P (atrial contraction); Q (downward deflection immediately before ventricular contraction); R (the peak of ventricular contraction); S (downward deflection immediately after ventricular contraction); and T (ventricular recovery). On the other hand, an atrial fibrillation wave registers irregularity, e.g., a P element is missing and a QRS element is irregular with no regular pattern. An example of the preprocessed ECG signal is given in Figure 1.

### 2.2. Deep Learning Models

**Figure 2:**
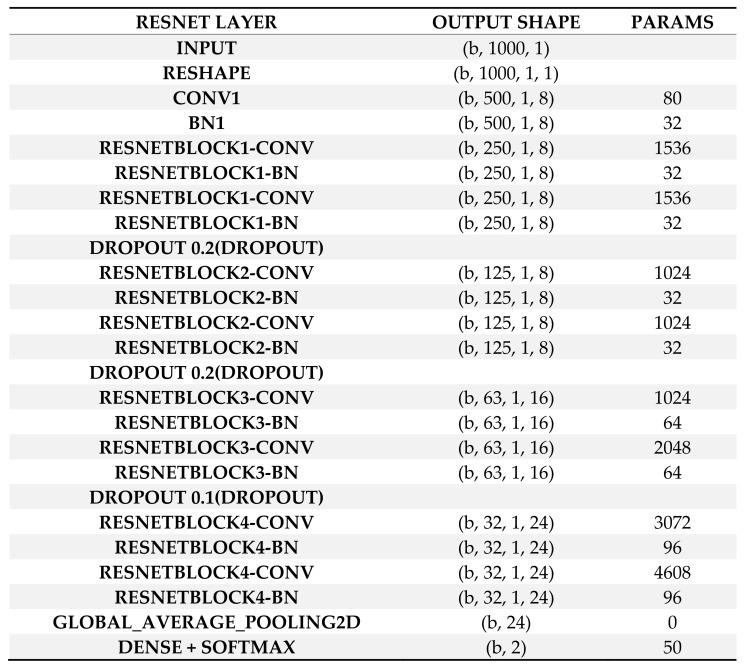
Resnet Architecture. Note: BN Batch Normalization, CONV Convolution, PARAMS Parameters.

**Figure 3:**
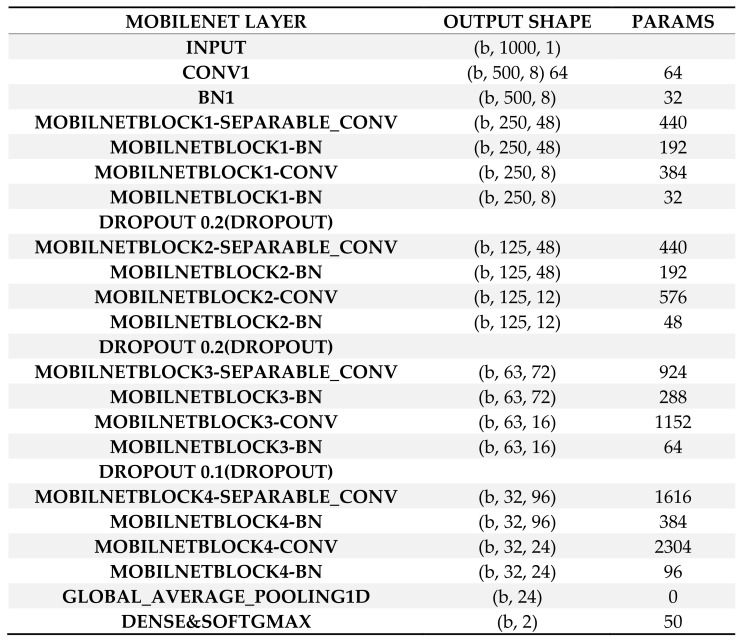
Mobilenet Architecture. Note: BN Batch Normalization, CONV Convolution, PARAMS Parameters.

For the diagnosis of arrhythmia in an embedded wearable device, this study applied and compared two neural network models, Resnet [21] and Mobilenet [22], with model compression in TensorFlow Lite [26]. The models used in this study are shown in Figure 2 and Figure 3. A neural network is a network of “neurons”, i.e., information units combined through weights. Usually, the neural network has one input layer, one, two or three intermediate layers and one output layer. Neurons in a previous layer connect with “weights” in the next layer and these weights represent the strengths of connections between neurons in a previous layer and their next-layer counterparts. This process starts from the input layer, continues through intermediate layers and ends in the output layer (feedforward operation). Then, learning happens: These weights are accommodated based on how much they contributed to the loss, a difference between the actual and predicted final outputs. This process starts from the output layer, continues through intermediate layers and ends in the input layer (backpropagation operation). The two operations are replicated until a certain expectation is met regarding the accurate diagnosis of the dependent variable. In other words, the performance of the neural network improves as long as its learning continues. Finally, a deep neural network is a neural network with a large number of intermediate layers, e.g., 5, 10 or even 1000. The deep neural network is called “deep learning” given that learning “deepens” through numerous intermediate layers [11,12].

Specifically, a certain type of deep learning models, so-called convolutional neural networks, have emerged as dominant deep learning models in the past decade. The convolutional neural network has convolutional layers, in which a kernel passes across input data and performs “convolution”, that is, computes the dot product of its own elements and their input-data counterparts. The operation of convolution helps the convolutional neural network to detect specific characteristics of the input data, e.g., the form of a normal rhythm vs. its arrhythmia counterpart. However, the convolutional neural network has an issue of gradient vanishing: As it becomes deeper (the number of its layers increases), the gradient of the loss with respect to the weight becomes 0 quickly. In this context, it has been an important task for deep learning experts to develop a new deep learning model, which manages its considerable depth (e.g., 100 layers) and unprecedented performance at the same time [11,12,21].

Resnet solved this great challenge based on residual learning explained below. This new deep learning model, which ranked first in 2015 ImageNet Large Scale Visual Recognition Challenge, was much deeper and more accurate than Virtual Geometry Group the second winner in 2014: the former network with 152 layers and top-5 error rate of 3.6% vs. the latter network with 24 layers and top-5 error rate of 6.8%. In its predecessor network, output *y* was the function of input *x*, i.e., *f*(*x*), whereas in Resnet, *y* is *f*(*x*) *+ x*. This helps to focus on “residual learning”, i.e., learning the residual part of *f*(*x*) besides *x*. In addition, this helps to overcome the gradient-vanishing problem: *f’*(*x*) *+* 1 > 1 [21]. Indeed, Mobilenet was presented as an efficient deep learning model for embedded vision applications: It is based on depth-wise and point-wise convolutions, which reduce the size of input image and the number of its channels, respectively [22].

Finally, TensorFlow Lite is a collection of tools for the compression and inference of an original TensorFlow model in an embedded device [26]. Once we complete the training of the original model, we can compress it in TensorFlow Lite (model compression) and we can run the inference of the compressed model in an embedded device. It is not an option in TensorFlow Lite to train a model at this point. The common strategies of model compression are pruning, quantization, clustering, low-rank approximation and knowledge distillation at this point [26,27,28] (Table 2). We use pruning to remove some of model weights, i.e., to set their values as zeroes (suitable for both training from scratch and using a pre-trained model) [29]. We use quantization to decrease the sizes of the weights by mapping their values in an original set to their smaller-set counterparts (e.g., 8-bit to 1-bit) (suitable for both training from scratch and using a pre-trained model) [30]. We use clustering to divide the weights into several groups, then share central values for all weights in the same group (suitable for both training from scratch and using a pre-trained model) [31]. We use low-rank approximation to reduce the redundancy (or “rank”) of convolutional filters, that is, to approximate the original filters based on their lower-rank counterparts (suitable for both training from scratch and using a pre-trained model). Finally, we use knowledge distillation to condense an original model to its smaller counterpart with a similar loss function (and performance) (suitable for using a pre-trained model) [32]. TensorFlow Lite supports pruning, quantization and clustering at this point [26].

## 3. Results

Firstly, Resnet and Mobilenet were compared in terms of six performance measures in this study, i.e., accuracy, sensitivity (or recall), specificity, area under the receiver-operating-characteristic curve (AUC), precision and F1 score. Their equations were presented as (1)–(5) below. Here, TP, FP, FN and TN represent true positive, false positive, false negative and true negative defined in a confusion matrix (Table 3).

(1) $$\mathrm{Accuracy}=\frac{\mathrm{TP}+\mathrm{TN}}{\mathrm{TP}+\mathrm{FN}+\mathrm{FP}+\mathrm{TN}}$$

(2) $$\mathrm{Sensitivity}/\mathrm{Recall}=\frac{\mathrm{TP}}{\mathrm{TP}+\mathrm{FN}}$$

(3) $$\mathrm{Specificity}=\frac{\mathrm{TN}}{\mathrm{FP}+\mathrm{TN}}$$

(4) $$\mathrm{Precision}=\frac{\mathrm{TP}}{\mathrm{TP}+\mathrm{FP}}$$

(5) $$F1\;\mathrm{Score}=\frac{2*\mathrm{Precision}*\mathrm{Recall}}{\mathrm{Precision}+\mathrm{Recall}}$$

(TP, FP, FN, TN true positive, false positive, false negative, true negative defined in Table 3)

**Figure 4:**
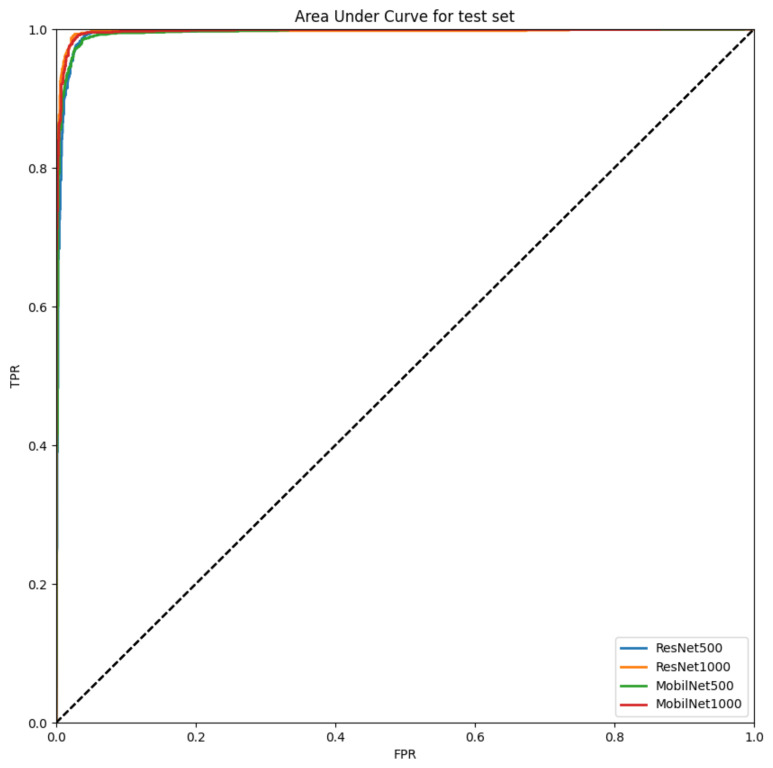
Area Under the Receiver-Operating-Characteristic Curve for the Test Set. Note: TPR True Positive Rate (Sensitivity), FPR False Positive Rate (1—Specificity).

Comparison was made between the original Resnet and its compressed counterpart in terms of the model weight size and performance (accuracy) in Table 4. The weight size of the compressed model registered a remarkable decrease from 743 MB to 76 KB (1/10,000), whereas its performance was almost the same as its original counterpart. In addition, a comparison was made between Resnet and Mobilenet in terms of the six performance measures in Table 5 and Figure 4. The two models were similar in terms of accuracy, i.e., Resnet-50 Hz (97.3) vs. Mobilenet-50 Hz (97.2), Resnet-100 Hz (98.2) vs. Mobilenet-100 Hz (97.9). Here, 50 Hz/100 Hz denotes the down-sampling rate. This similarity remained intact in terms of the other performance measures as well: e.g., 98.1 vs. 97.7 (F1 score), 99.1 vs. 98.3 (sensitivity/recall), 97.5 vs. 97.6 (specificity), 97.1 vs. 97.2 (precision), and 99.6 vs. 99.7 (AUC) regarding Resnet-100 Hz vs. Mobilenet-100 Hz for the test set.

**Figure 5:**
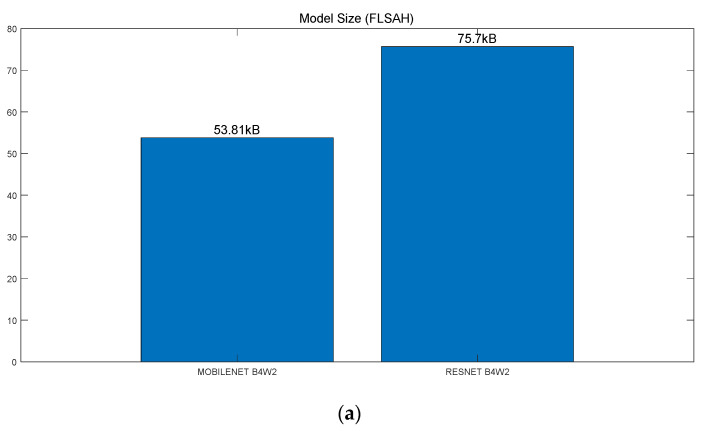

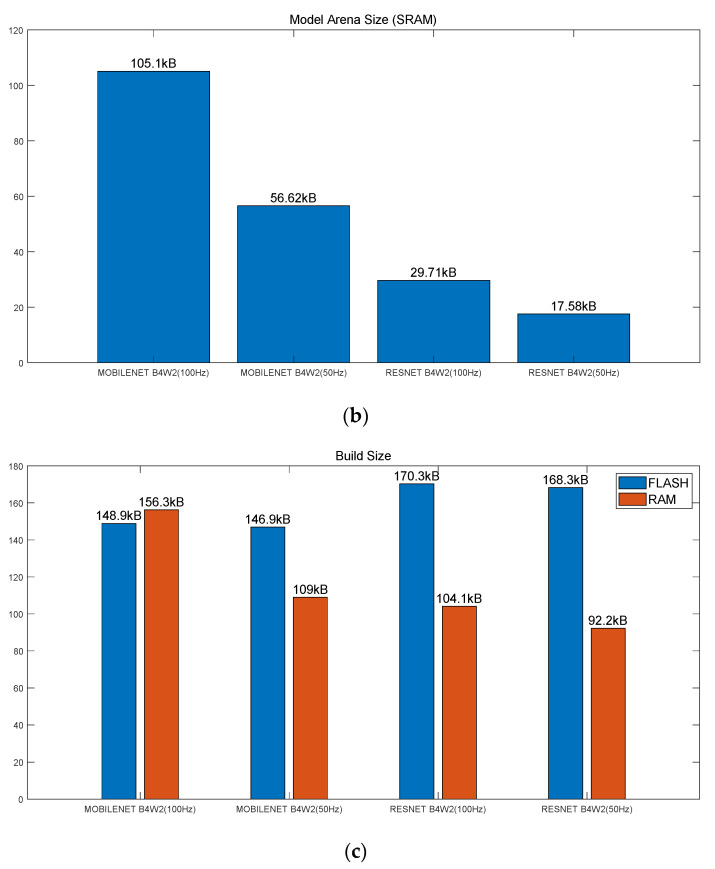
Model Size (**a**) Model Size (FLASH) (**b**) Model Arena Size (SRAM) (**c**) Model Build Size.

**Figure 6:**
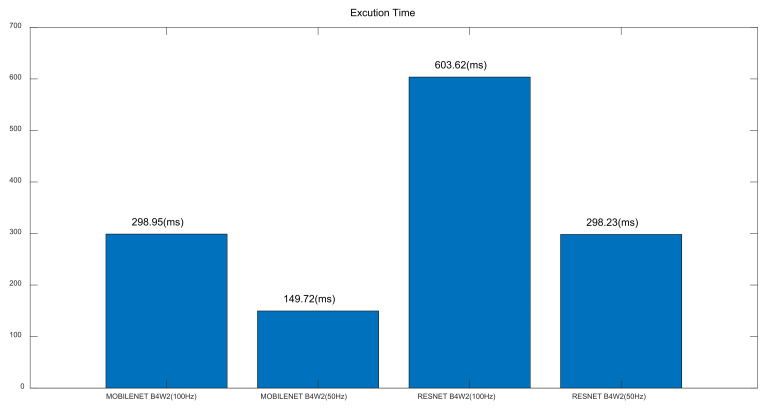
Execution Time.

**Figure 7:**
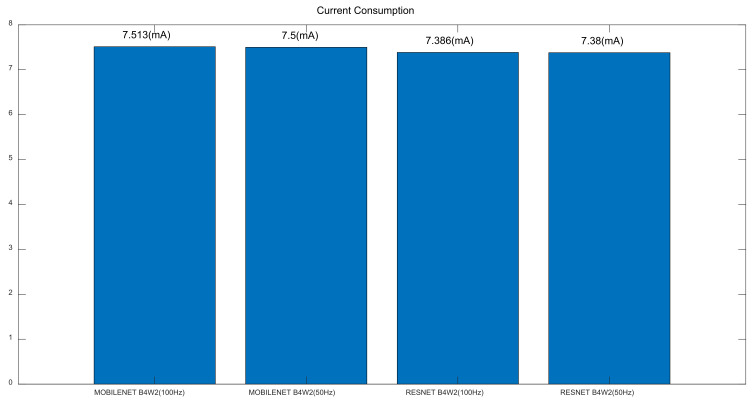
Current Consumption.

Secondly, Resnet and Mobilenet were compared in terms of model size (FLASH), model arena size (SRAM) and model build size in Figure 5. The former model took more flash memory in Figure 5c: Resnet-50 Hz (168.3 KB) vs. Mobilenet-50 Hz (146.9 KB), Resnet-100 Hz (170.3 KB) vs. Mobilenet-100 Hz (148.9 KB). However, the opposite was true for random access memory in Figure 5c: Resnet-50 Hz (92.2 KB) vs. Mobilenet-50 Hz (109.0 KB), Resnet-100 Hz (104.1 KB) vs. Mobilenet-100 Hz (156.3 KB) (Figure 5c). Thirdly, it was shown in Figure 6 that Resnet took longer inference time than Mobilenet: Resnet-50 Hz (298.23 ms) vs. Mobilenet-50 Hz (149.72 ms), Resnet-100 Hz (603.62 ms) vs. Mobilenet-100 Hz (298.95 ms) (Figure 6). Fourthly, current consumption was reported to be similar among the four models, i.e., Resnet-50 Hz (7.4 mA), Mobilenet-50 Hz (7.5 mA), Resnet-100 Hz (7.4 mA), Mobilenet-100 Hz (7.5 mA) (Figure 7). Overall, Mobilenet would be a more efficient model than Resnet to classify arrhythmia in an embedded wearable device.

## 4. Discussion

### 4.1. Contributions of Study

The emerging literature has focused on the early diagnosis of arrhythmia, using deep neural networks for better performance measures than those of other approaches. These studies utilized ECG data, applying convolutional neural networks, recurrent neural networks or both with various class categories and accuracy results. However, these models are reported to take too much memory for an embedded wearable device. The importance of an embedded wearable device with automatic detection and alarming cannot be overstated, given that 15–30% of patients with atrial fibrillation are reported to be asymptomatic. These asymptomatic patients do not seek medical care, hence traditional diagnostic tools including Holter are not effective for the further prevention of associated stroke or heart failure. This is likely to be more so in the era of COVID-19, in which patients become more reluctant on hospitalization and checkup. However, little literature is available on this important topic. For this reason, this study developed efficient deep learning with model compression, which is designed to use ECG data and classify arrhythmia in an embedded wearable device.

**Figure 8:**
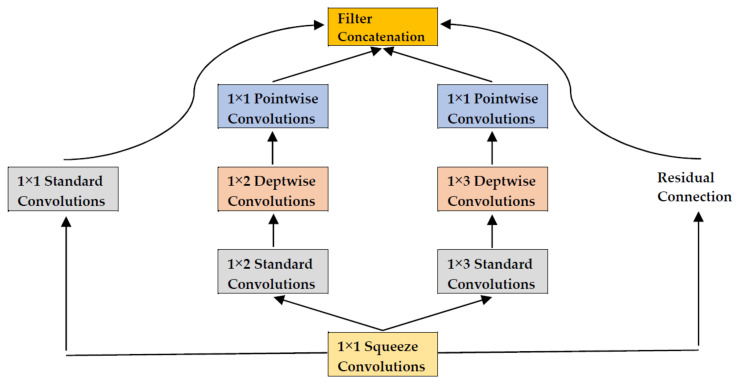
Lite Module. Source: [23].

A rare attempt was made to use a “lightweight” convolutional neural network (Litenet) for the classification of arrhythmia and achieved the accuracy of 97.78% in the inference time of 25 microsecond [23]. Here, the term “lightweight” means the size of input image and/or the number of its channels were reduced as in Mobilenet. The core of Litenet is the Lite module, a modified version of the inception module with two distinctive characteristics, i.e., (1) the kernel sizes of 1 × 1, 1 × 2 and 1 × 3 and (2) depth-wise and point-wise convolutions, which reduce the size of the input image and the number of its channels (Figure 8). ECG data for this study came from the MIT-BIH Arrhythmia Database with 109,449 samples from 48 unique participants. These samples were augmented and oversampled to achieve a balance between normal and arrhythmia categories. Then, five deep learning models were compared in terms of accuracy and inference time: Alexnet, Googlenet, Litenet, Mobilenet and Squeezenet. Litenet ranked third in accuracy and first in inference time.

**Figure 9:**
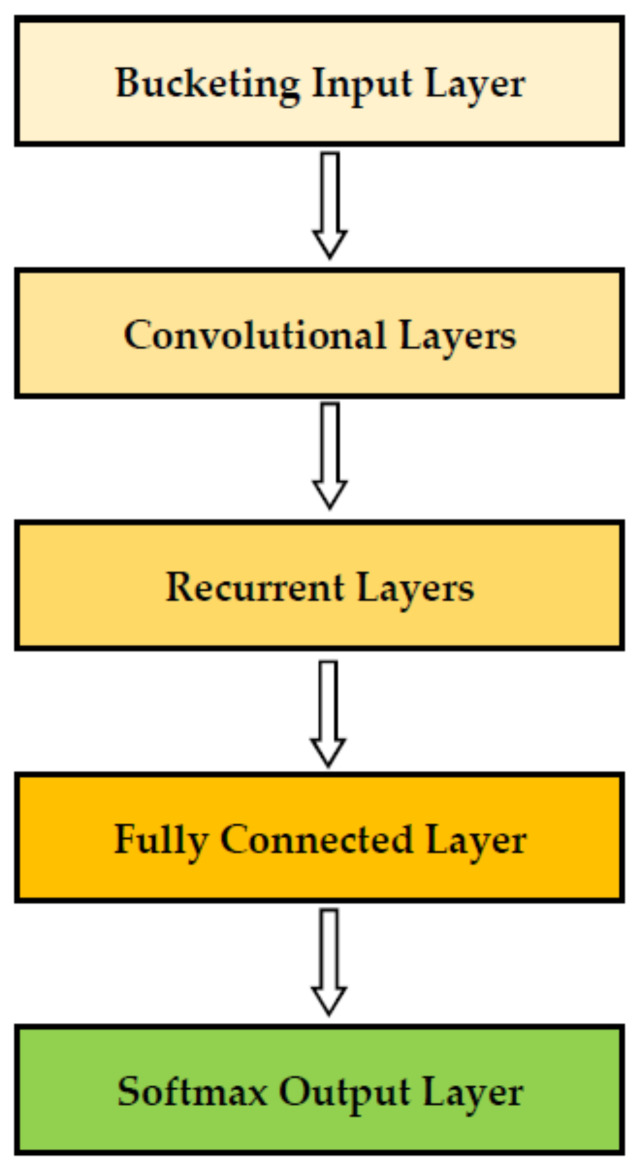
Fused Lightweight Recurrent Neural Network. Source: [25].

Another study employed a lightweight recurrent neural network for the diagnosis of arrhythmia and recorded the accuracy of 99.80% in the inference time of 3 min [25]. This study developed the fused lightweight recurrent neural network module: combination of convolutional layers and the simplest (Vanilla) recurrent layers to achieve efficiency and accuracy at the same time (Figure 9). ECG data for this study also came from the MIT-BIH Arrhythmia Database with 48 unique participants. Their samples were undersampled to achieve a balance between normal and arrhythmia categories. However, these studies relied on a public dataset (MIT-BIH Arrhythmia Database) and their inference was carried out in personal computers, not in an embedded wearable device. For this reason, this study developed efficient deep learning with model compression, which is designed to use ECG data and classify arrhythmia in an embedded wearable device. To the best of our knowledge, this is the first study in this direction.

### 4.2. Limitations of Study

First, this study used the binary categories of normal vs. arrhythmia conditions. Introducing the multiple categories of arrhythmia would be a great extension of research on this topic. Secondly, little literature is available, and more study is to be done regarding the comparison of convolutional neural networks and their recurrent counterparts in terms of model compression, model performance and inference time. As addressed above, the convolutional neural network has convolutional layers, in which a kernel passes across input data and performs “convolution”, that is, computes the dot product of its own elements and their input-data counterparts. The operation of convolution helps the convolutional neural network to detect specific characteristics of the input data, e.g., the form of a normal rhythm vs. its arrhythmia counterpart. On the other hand, in the recurrent neural network, the current output information depends, in a repetitive (or “recurrent”) pattern, on the current input information and the previous hidden state (which is the memory of the network on what happened in all previous periods) [24,25,33]. In other words, the convolutional neural network focuses on global information whereas its recurrent counterpart focuses on sequential information. Combining these unique strengths is expected to render great insights and rich applications for the field of efficient deep learning with model compression. To the best of our knowledge, however, no study has been completed in this direction.

Thirdly, the standardization of ECG diagnostic criteria would strengthen the agreement of clinical experts and the performance of computer algorithms regarding ECG interpretation [31]. Clinical experts with rich experience, the gold standard, often disagree in their ECG interpretation, hence, more endeavor is to be made in this direction. Finally, this study did not consider the application of reinforcement learning to find the most efficient deep learning models [32,33] for the classification of arrhythmia in an embedded wearable device. Reinforcement learning helps to find the optimal deep learning model with the best performance in an embedded wearable device, given the budget constraint of model size, inference time, current consumption and so on as in this study [32,33]. This study considered Resnet and Mobilenet to overcome the issue of gradient vanishing and to manage considerable depth and best performance in an embedded wearable device, given the budget constraint of model size, inference time and current consumption. These two models were chosen largely because there have been few options available. However, various deep learning models can be developed with different sets of metrics including performance, model size, inference time and current consumption. How to optimize the deep learning model in an embedded wearable device given the constraint of various metrics is still an uncharted territory and much more research is to be completed for this emerging field.

## 5. Conclusions

Little literature is available on compressed deep learning to classify arrhythmia in an embedded wearable device. In this context, this study introduced efficient deep learning with model compression, which is tailored for ECG data and arrhythmia classification in an embedded wearable device. To the best of our knowledge, this is the first study in this direction. Based on the results of this study, Mobilenet would be a more efficient model than Resnet to classify arrhythmia in an embedded wearable device.

## Figures and Tables

**Table 1:** Categories: Normal vs. Arrhythmia.

Category	Diagnosis	Count
1	Normal Sinus Rhythm	9760
2	Sinus Bradycardia	1944
3	Sinus Tachycardia	1754
4	First-Degree Atrioventricular Block	1954
5	Premature Ventricular Contraction	1566
6	Atrial Fibrillation	9584
7	Atrial Flutter	1746
	Total	28,308

Note: Normal [1,2,3,4] vs. Arrhythmia [5,6,7].

**Table 2:** Model Compression for Deep Learning.

Approach	Explanation
Pruning	We use pruning to remove some of model weights, i.e., to set their values as zeroes: suitable for both training from scratch and using a pre-trained model [29]
Quantization	We use quantization to decrease the sizes of the weights by mapping their values in an original set to their smaller-set counterparts (e.g., 8-bit to 1-bit): suitable for both training from scratch and using a pre-trained model [30]
Clustering	We use clustering to divide the weights into several groups, then share central values for all weights in the same group: suitable for both training from scratch and using a pre-trained model [31]
Low-Rank Approximation	We use low-rank approximation to reduce the redundancy (or “rank”) of convolutional filters, that is, to approximate the original filters based on their lower-rank counterparts: suitable for both training from scratch and using a pre-trained model
Knowledge Distillation	We use knowledge distillation to condense an original model to its smaller counterpart with a similar loss function (and performance): suitable for training from scratch [32]

**Table 3:** Confusion Matrix.

		True	
		*Arrhythmia*	*Normal*
**Predicted**	*Arrhythmia*	TP (True Positive)	FP (False Positive)
	*Normal*	FN (False Negative)	TN (True Negative)

**Table 4:** Original vs. Compressed Deep Learning: Model Weight Size, Accuracy and Inference Time.

	Cloud Version	Embedded Version
Model Weight Size	743 MB	**76 KB**
Accuracy	98.4%	**97.2%**
Inference Time	NA	**<298 ms**

**Table 5:** Model Performance.

**Model**	**Resnet with 50 Hz Sampling Rate**			**Resnet with 100 Hz Sampling Rate**		
**Class**	Metric	Validation Set	Test Set	Metric	Validation Set	Test Set
**Positive:** **Arrhythmia** **Negative: Normal**	Acc	0.9735	0.9728	Acc	0.9802	0.9823
	F1	0.9721	0.9706	F1	0.9791	0.9808
	Sensitivity	0.9660	0.9868	Sensitivity	0.9850	0.9907
	Specificity	0.9820	0.9611	Specificity	0.9760	0.9753
	Precision	0.9837	0.9550	Precision	0.9733	0.9711
	AUC	0.9932	0.9937	AUC	0.9964	0.9963
**Model**	**Mobilenet with 50 Hz Sampling Rate**			**Mobilenet with 100 Hz Sampling Rate**		
**Class**	Metric	Validation Set	Test Set	Metric	Validation Set	Test Set
**Positive:** **Arrhythmia** **Negative: Normal**	Acc	0.9675	0.9717	Acc	0.9785	0.9792
	F1	0.9656	0.9694	F1	0.9772	0.9773
	Sensitivity	0.9692	0.9822	Sensitivity	0.9812	0.9829
	Specificity	0.9660	0.9630	Specificity	0.9760	0.9760
	Precision	0.9620	0.9569	Precision	0.9732	0.9716
	AUC	0.9942	0.9908	AUC	0.9945	0.9967

Note: Acc Accuracy, AUC Area Under the Receiver-Operating-Characteristic Curve.

## Data Availability

The datasets used and/or analyzed during the current study are available from the corresponding author on reasonable request.

## References

[1] World Health Organization Cardiovascular Diseases (CVDs). https://www.who.int/news-room/fact-sheets/detail/cardiovascular-diseases-(cvds).

[2] Chugh S.S., Havmoeller R., Narayanan K., Singh D., Rienstra M., Benjamin E.J., Gillum R.F., Kim Y.-H., McAnulty J.H., Zheng Z.J. (2014). Worldwide epidemiology of atrial fibrillation: A Global Burden of Disease 2010 Study. Circulation.

[3] Statistics Korea (2021). Year 2020 Statistics on Causes of Death in Korea.

[4] Kim Y.E., Park H., Jo M.W., Oh I.H., Go D.S., Jung J., Yoon S.J. (2019). Trends and patterns of burden of disease and injuries in Korea using disability-adjusted life years. J. Korean Med. Sci..

[5] Kim D., Yang P.S., Jang E., Yu H.T., Kim T.H., Uhm J.S., Kim J.Y., Sung J.H., Pak H.N., Lee M.H. (2018). Increasing trends in hospital care burden of atrial fibrillation in Korea, 2006 through 2015. Heart.

[6] Isin A., Ozdalili S. (2017). Cardiac arrhythmia detection using deep learning. Procedia Comput. Sci..

[7] Rajpurkar P., Hannun A.Y., Haghpanahi M., Bourn C., Ng A.Y. (2017). Cardiologist-level arrhythmia detection with convolutional neural networks. arXiv Prepr..

[8] Li D., Zhang J., Zhang Q., Wei Z. Classification of ECG signals based on 1d convolutional neural network. Proceedings of the 2017 IEEE 19th International Conference on e-Health Networking, Applications and Services (Healthcom).

[9] Sannino G., De Pietro G. (2018). A deep learning approach for ECG-based heartbeat classification for arrhythmia detection. Future Gener. Comput. Syst..

[10] Jun T.J., Nguyen H.M., Kang D., Kim D., Kim D., Kim Y.H. (2018). ECG arrhythmia classification using a 2-D convolutional neural network. arXiv Prepr..

[11] Lee K.S., Jung S., Gil Y., Son H.S. (2019). Atrial fibrillation classification based on convolutional neural networks. BMC Med. Inform. Decis. Mak..

[12] Park J., Kim J.-K., Jung S., Gil Y., Choi J.-I., Son H.S. (2020). ECG-signal multi-classification model based on squeeze-and-excitation residual neural networks. Appl. Sci..

[13] Zhang C., Wang G., Zhao J., Gao P., Lin J., Yang H. Patient-specific ECG classification based on recurrent neural networks and clustering technique. Proceedings of the 2017 13th IASTED International Conference on Biomedical Engineering (BioMed).

[14] Kim K. Arrhythmia Classification in Multi-Channel ECG Signals Using Deep Neural Networks. http://www2.eecs.berkeley.edu/Pubs/TechRpts/2018/EECS-2018-80.html.

[15] Levy S., Maarek M., Coumel P., Guize L., Lekieffre J., Medvedowsky J.L., Sebaoun A. (1999). Characterization of different subsets of atrial fibrillation in general practice in France: The ALFA study. The college of French cardiologists. Circulation.

[16] Flaker G.C., Belew K., Beckman K., Vidaillet H., Kron J., Safford R., Mickel M., Barrell P. (2005). Asymptomatic atrial fibrillation: Demographic features and prognostic information from the atrial fibrillation follow-up investigation of rhythm management (AFFIRM) study. Am. Heart J..

[17] Kerr C., Boone J., Connolly S., Greene M., Klein G., Sheldon R., Talajic M. (1996). Follow-up of atrial fibrillation: The initial experience of the Canadian registry of atrial fibrillation. Eur. Heart J..

[18] Rienstra M., Lubitz S.A., Mahida S., Magnani J.W., Fontes J.D., Sinner M.F., Van Gelder I.C., Ellinor P.T., Benjamin E.J. (2012). Symptoms and functional status of patients with atrial fibrillation: State of the art and future research opportunities. Circulation.

[19] Czeisler M.É., Marynak K., Clarke K.E.N., Salah Z., Shakya I., Thierry J.M., Ali N., McMillan H., Wiley J.F., Weaver M.D. (2020). Delay or avoidance of medical care because of COVID-19-related concerns—United States, June 2020. Morb. Mortal. Wkly. Rep..

[20] Sun C., Dyer S., Salvia J., Segal L., Levi R. (2021). Worse cardiac arrest outcomes during The COVID-19 pandemic in Boston can be attributed to patient reluctance to seek care. Health Aff..

[21] He K., Zhang X., Ren S., Sun J. (2017). Deep residual learning for image recognition. arXiv Prepr..

[22] Howard A.G., Zhu M., Chen B., Kalenichenko D., Wang W., Weyand T., Andreetto M., Adam H. (2017). MobileNets: Efficient convolutional neural networks for mobile vision applications. arXiv Prepr..

[23] He Z., Zhang X., Cao Y., Liu Z., Zhang B., Wang X. (2018). LiteNet: Lightweight neural network for detecting arrhythmias at resource-constrained mobile devices. Sensors.

[24] Saadatnejad S., Oveisi M., Hashemi M. (2020). LSTM-based ECG classification for continuous monitoring on personal wearable devices. IEEE J. Biomed. Health Inform..

[25] Jeon E., Oh K., Kwon S., Son H., Yun Y., Jung E.S., Kim M.S. (2020). A lightweight deep learning model for fast electrocardiographic beats classification with a wearable cardiac monitor: Development and validation study. JMIR Med. Inform..

[26] TensorFlow Lite Model Optimization. https://www.tensorflow.org/lite/performance/model_optimization.

[27] Cheng Y., Wang D., Zhou P., Zhang T. (2017). A Survey of Model Compression and Acceleration for Deep Neural Networks. arXiv Prepr..

[28] Lee Y.J., Moon Y.H., Park J.Y., Min O.G. (2019). Recent R&D trends for lightweight deep learning. Electron. Telecommun. Trends.

[29] Han S., Mao H., Dally W.J. (2015). Deep Compression: Compressing Deep Neural Networks with Pruning, Trained Quantization and Huffman Coding. arXiv Prepr..

[30] Rastegari M., Ordonez V., Redmon J., Farhadi A. XNOR-Net: ImageNet Classification Using Binary Convolutional Neural Networks. https://arxiv.org/abs/1603.05279.

[31] Ullrich K., Meeds E., Welling M. (2017). Soft Weight-Sharing for Neural Network Compression. arXiv Prepr..

[32] Hinton G., Vinyals O., Dean J. (2015). Distilling the Knowledge in a Neural Network. arXiv Prepr..

[33] Lee K.S., Park K.W. (2019). Social determinants of the association among cerebrovascular disease, hearing loss and cognitive impairment in a middle-aged or older population: Recurrent neural network analysis of the Korean Longitudinal Study of Aging (2014–2016). Geriatr. Gerontol. Int..

